# How do the determinants of exclusive breast-feeding change over time? A multi-survey quasi-longitudinal study in Lao People’s Democratic Republic

**DOI:** 10.1017/S1368980022001380

**Published:** 2022-09

**Authors:** Niels Bal, Sayvisene Boulom, Kimberly A van Brakel, Sengchanh Kounnavong, Dirk R Essink

**Affiliations:** 1 Department of Health Sciences, Faculty of Science, Vrije Universiteit, De Boelelaan 1105, 1081 HV Amsterdam, Netherlands; 2 Rural Economic and Food Technology Department, Faculty of Agriculture, National University of Laos, Vientiane, Lao PDR; 3 Athena Institute, Faculty of Science, Vrije Universiteit, Amsterdam, Netherlands; 4 Lao Tropical and Public Health Institute, Ministry of Health, Vientiane, Lao PDR

**Keywords:** Exclusive breast-feeding, Lao People’s Democratic Republic, Longitudinal, Multiple indicator cluster survey, Nutrition

## Abstract

**Objective:**

The current study aimed to assess trends, associated factors and the changes in these factors for exclusive breast-feeding (EBF) over the past two decades in Lao People’s Democratic Republic (Lao PDR).

**Design:**

The current study used a quasi-longitudinal design. Descriptive analyses were done with correction for complex survey design. Inferential analyses were done for survey years separately using multiple logistic regression. Finally, pooled logistic regression analysis was done using interaction terms to quantify the difference in association per year.

**Setting:**

The current study used data from all provinces of Lao PDR collected in the years 2000, 2006, 2011/2012 and 2017.

**Participants:**

Children aged six months or younger from Lao PDR.

**Results:**

EBF practice was estimated at 19·03 %, 26·87 %, 40·67 % and 44·89 % in the four survey years, respectively. Factors significantly associated with EBF included: region of residence, ethnicity, wealth index and age of child. Region and ethnicity saw significant changes in association, and the South developing positively over time as well as in the Lao-Thai ethnic group. Having had any antenatal visits was not associated with EBF practice, nor did this change over time.

**Conclusions:**

Our study shows how EBF trends, and factors associated with EBF, changed over time. We applied an easily replicable methodology to assess similar public health phenomena. We argue that such analysis is particularly relevant for transitioning countries. In such rapidly evolving settings, it is crucial to take into account changing underlying factors when assessing and developing public health policy.

During the past decades, large-scale data collection has become increasingly common in epidemiological research and global health. Examples of these large-scale collections are the Demographic Health Survey and equivalents such as the Multiple Indicator Cluster Survey (MICS), aimed at measuring the results of efforts made on public health issues with respect to the Millennium Development Goals and Sustainable Development Goals. One such issue is exclusive breast-feeding (EBF), one of the most important practices for infant development and survival, as breastmilk contains all the necessary nutrients for the first years of the infant’s life^(1,2)^.

The WHO recommends that all children receive EBF for the first six months of their life^(3)^. Most infant deaths occur in these six months, especially in lower-income countries; this is the most neglected period for adequate quality of care^(3–5)^. The worldwide rate of EBF was estimated at 40·06 % in 2014, with the highest rates found mostly in countries on the African continent^(6–9)^. Although the global rate of EBF has been rising, the increase has stagnated in recent years and is still falling short of the global goal of 50 % coverage by 2025^(3,7,10–12)^. If EBF rates were to be scaled up to near-universal levels, an estimated 823 000 deaths of children under five (CU5) could be prevented annually^(6,13)^. Lack of EBF is associated with lower infant intelligence in later life, and with estimated annual global economic losses of $302 billion, or 0·49 % of the worldwide Gross National Income^(6,13)^. Considering the benefits of EBF, it should be one of the most intelligent and cost-effective stimuli for economic growth and human capital^(14)^. Considering the evident benefits, many efforts have been made to increase EBF rates, such as the Baby-Friendly Hospital Initiative and WHO Code of Marketing of Breast Milk Substitutes (The Code)^(10,15)^.

The factors that determine whether a mother practices EBF have been the subject of extensive research. The conceptual framework adopted by UNICEF, which indicates that EBF is a multi-dimensional concern, is a result of such research^(6,13)^. The framework stipulates that the practice of EBF is not a one-woman job, but requires an enabling environment, support from the father and adequate government policy, among other factors^(6,13)^. Many of these factors, such as maternal employment, antenatal care (ANC) and ethnicity, have been identified through research in different study settings which has provided input for the formulation of health policy^(16–22)^.

While most of the associations are described in relation to locations and contexts, how they change over time is often unknown, as they are often based on cross-sectional study results. Given the adaptive and thus fluctuating nature of human behaviour, the assumption that the associations remain static over time is highly unlikely and therefore naive. Additionally, to determine whether policy is successful in improving EBF, trends and temporal associations should be assessed. This is especially important for transitioning countries that experience significant developments from both an economic and a human capital point of view. A few studies have already shown that associations can appear, disappear or change over time^(21,23–25)^. For example, Santos *et al*. showed that the inequalities in breast-feeding between poorer and wealthier mothers from Pelotas, Brazil, changed over time and that EBF adaptation was picked up more rapidly among the wealthier mothers^(25)^.

The few studies that assessed these changes over time did so with a range of different methodologies. For example, more qualitative interpretations of the study results were used, when comparing the prevalence of EBF over several years and/or among the factors studied^(24,25)^. In addition, such rates can be statistically tested using trend tests, but they lack the benefit of measuring effect sizes and thus direction and magnitude. While there are studies that statistically quantify changes between years with respect to the magnitude and direction of associations, they remain scarce, as does research investigating which statistical methodologies are most applicable^(21,23)^.

One transitioning country lacking temporal analysis of EBF trends and associated factors is Lao People’s Democratic Republic (Lao PDR). Similar to the global trend of EBF, Lao PDR has seen improvements in the past two decades, but these improvements have stagnated in the more recent years^(26,27)^. EBF has important implications for Lao PDR, as the country still faces a large burden of stunting among CU5 as a result of chronic malnutrition^(28)^. In order to support maintaining the momentum in EBF improvement, the current study aims to investigate the trends, associated factors and the changes in these factors for EBF over the past two decades in Lao PDR, in an effort to contribute to the development of robust and appropriately designed policies and programmes. We have applied an easily replicable statistical methodology which quantifies change over time, using the rich data available from the MICS and Lao Social Indicator Survey (LSIS) data banks in Lao PDR.

## Method

### Study design and sampling

The current study used a quasi-longitudinal study design, allowing for a time-efficient and simple comparison of multiple large-scale survey data in a temporal fashion. Data were derived from four large-scale representative survey samples of the Lao population: the MICS of 2000 and 2006 and the LSIS of 2011/2012 and 2017.

The national surveys selected their original samples with a probability proportional estimation size. These estimations were based on a list of area units with the latest estimates of the household sizes from the latest agricultural census^(29)^. A two-stage sampling strategy was used, the first of which consisted of selecting villages as primary sampling units/clusters. These primary sampling units were selected proportional to the distribution of urban, rural and rural without road proportions present in each of the three regions based on the most recent census frame available. The combination of the urban rural distribution and the regions were used as the sampling strata in the MICS 2000 survey, resulting in six strata. In the following year, a similar division was used with the addition of rural with/without road, resulting in nine strata. With the introduction of the LSIS, a more elaborate method was used by using urban, rural with/without road in combination with the seventeen provinces in 2011/2012 and eighteen provinces in 2017, resulting in fifty-one and fifty-four strata, respectively. The second stage entailed the sampling of households within each village, by means of standard systematic sampling^(30)^. Finally, sampling weights in the samples were based on the PSU and are the inverse probabilities of individuals selected in the survey.

### Inclusion and exclusion

Primary criteria were data recorded on the outcome variable and age between 0 and five months. The age gaps included children that ranged from 0 to six months (excluding the latter), indicating that the true range was from the moment of birth to five months and 28, 29, 30 or 31 d depending on the month and year. Any children for whom data on their mother/caretaker were not available were also excluded from the analyses. Finally, twins were also excluded from the analyses. Selected predictor variables, their value labels and availability across the data sets can be found in the supplementary file.

### Data aggregation

Across all data sets, different codes were used for similar variables. Data were merged on the basis of these unique identifiers: cluster number, household number and caretaker line number. The merge strategy used was a one-to-many merge, as caretaker variables could apply to multiple children (some caretakers/mothers had more than one CU5).

### Variables of interest

The outcome variable for EBF was a dichotomous variable constructed using the following conditions: whether the woman was breast-feeding at the time of the survey and whether she had given the infant any food (besides vitamin supplements and/or ORS) besides her breastmilk within the 24 h preceding the survey. Thus, the variable was coded to be 0 = the mother/caretaker gives anything else besides breast-feeding, or 1 = the mother/caretaker exclusively feeds her infant breastmilk (may include vitamin supplements and/or ORS). The variables used for the study can be found in Table 1.

**Table 1:** Sample characteristics of children aged 0–5 months per survey year

	MICS 2000		MICS 2006		LSIS 2011/12		LSIS 2017	
	*n*	%	*n*	%	*n*	%	*n*	%
Age of caretaker in years								
15–19	82	14·95	81	18·29	192	16·72	163	14·70
20–24	161	29·31	122	27·46	387	33·79	331	29·93
25–29	126	22·96	115	25·89	266	23·25	322	29·09
30–34	88	16·10	68	15·43	159	13·86	165	14·87
35–39	67	12·29	37	8·30	95	8·33	91	8·26
40–44	24	4·38	21	4·63	46	4·05	35	3·15
Caretaker currently in union								
Yes	521	94·85	–		1122	97·97	1083	97·82
No	28	5·15	–		23	2·03	24	2·18
Education of caretaker*								
No education	232	42·64	169	38·44	331	28·88	204	18·41
Primary	232	42·64	179	40·71	447	39·01	434	39·21
Secondary	80	14·17	92	20·85	368	32·10	469	42·38
Reading ability of caretaker								
Cannot read	228	41·96	211	60·80	454	58·61	333	52·20
Difficulty reading	57	10·60	41	11·67	124	16·02	130	20·36
No problem	267	47·44	96	27·53	197	25·37	175	27·44
Wealth index								
Poorest	160	29·09	132	29·75	279	24·38	310	28·00
Poor	97	17·70	112	25·19	267	23·30	220	19·92
Middle	97	17·59	73	16·44	230	20·05	203	18·35
Rich	102	18·64	55	12·47	194	16·90	184	16·59
Richest	93	16·98	72	16·14	176	15·36	190	17·14
Gender of household head								
Male	517	94·16	412	92·86	1039	90·73	980	88·55
Female	32	5·84	32	7·14	106	9·27	127	11·45
Ethnicity of household head								
Lao-Thai	241	43·95	211	47·69	552	48·17	599	54·14
Mon-Khmer	82	14·95	55	12·50	120	10·47	302	27·26
Hmong-Mien	52	9·40	79	17·90	157	13·58	159	14·35
Other groups	174	31·71	97	21·90	318	27·78	47	4·24
Religion of household head								
Buddhist	–		222	49·97	664	58·04	609	55·01
Animist	–		–		463	40·54	482	43·51
Mother	–		10	2·25	16	1·42	16	1·48
No religion	–		212	47·78	–		–	
Frequency of watching TV of caretaker								
Not at all	–		–		347	30·25	325	29·36
Less than once a week	–		–		54	4·76	41	3·74
Once a week	–		–		128	11·17	118	10·66
Almost everyday	–		–		616	53·82	622	56·25
Region								
North	182	33·08	164	37·02	351	30·68	365	32·94
Central	176	32·06	180	40·60	532	46·41	497	44·87
South	191	34·86	99	22·38	262	22·91	246	22·19
Residence								
Urban	105	19·15	74	16·67	230	20·10	279	25·22
Rural	444	80·85	369	83·33	915	79·90	828	74·78
Caretaker received ANC								
Yes	137	30·64	181	40·77	658	57·93	914	84·14
No	311	69·36	263	59·23	478	42·07	172	15·86
Caretaker times received ANC								
0	–		–		478	42·14	172	15·90
1	–		–		64	5·63	50	4·60
2	–		–		72	6·37	66	6·14
3	–		–		104	9·15	125	11·54
4	–		–		108	9·47	145	13·35
< 4	–		–		309	27·23	525	48·77
Caretaker practiced food taboo†								
Yes	464	84·78	354	81·57	–		–	
No	83	15·22	80	18·43	–		–	
Child age in months								
0	54	9·88	42	9·52	229	19·97	199	17·96
1	91	16·63	91	20·51	209	18·26	157	14·24
2	129	23·52	66	14·99	211	18·39	198	17·88
3	97	17·67	89	20·09	171	14·91	178	16·12
4	95	17·25	85	19·23	166	14·50	196	17·69
5	83	15·05	69	15·66	160	13·96	178	16·11
Gender of child								
Male	267	48·50	217	48·96	563	49·12	554	50·02
Female	283	51·50	226	51·04	583	50·88	553	49·98
Place of birth								
Home	–		322	74·20	645	56·77	358	33·00
Facility	–		112	25·80	491	43·23	727	67·00
Child had diarrhoea in the preceding 2 weeks?								
Yes	35	6·44	42	9·42	98	8·57	70	6·33
No	513	93·56	402	90·58	1047	91·43	1035	93·66
Children ever born by caretaker								
1	–		–		391	34·30	360	32·75
2	–		–		267	23·38	299	27·24
3	–		–		173	15·13	199	18·11
4	–		–		93	8·13	125	11·41
< 4	–		–		218	19·06	115	10·49
Time put baby to breast								
Immediately	165	31·71	116	27·04	410	36·92	515	48·10
Hours	194	37·45	110	25·49	380	34·21	372	34·74
Days	160	30·85	204	47·47	320	28·87	184	17·16
Child has vaccination card‡								
Yes	170	30·77	205	46·24	591	51·57	856	77·40
No	380	69·23	238	53·76	555	48·43	250	22·60

Sample size per variable found in supplementary file.

*Primary education consists of 5 years (grades 1 to 5), secondary education consist of 7 years (grades 1 to 7).

† A food taboo is a traditional practice in which women that recently gave birth abstain of eating certain foods. These practices vary greatly between ethnic groups.

‡ A card not seen, but indicated as ‘yes’ by the mother was considered ‘no’.

### Data analysis

Analysis of the data was carried out using descriptive and inferential statistics. Recoding and aggregation of data was done using IBM SPSS statistics version 25^(31)^. Descriptive and inferential statistics were further carried out using STATA se version 16^(32)^.

### Descriptive statistics

For the descriptive analysis of the study, correction for the complex survey designs was done in order to provide valid estimates of population parameters such as the proportion of EBF per survey year. These corrections were done using the ‘svyset’ function in STATA SE. By indicating the various components in the module, such as the sampling weights, primary sampling units and sampling strata, further estimates could be done per survey. Within the survey design corrections, sampling weights were used. For the descriptive statistics, variance estimation was done employing the commonly used Taylor-linearisation method, which is computationally efficient^(33)^. Variance estimation was done using the entire population of each individual survey sample. Every analysis that was done using the complex survey design correction was carried out on the pre-specified subpopulation, based on the discussed in/exclusion criteria. Design efficiency was assessed with the design effect, which is the ratio between the actual estimated variance based on the design and the variance of the same sample regarded as a simple random sample (SRS)^(34)^. Any singleton units (clusters having only one observation) were marked as certainty units in the design correction, indicating that they did not contribute to variance estimation. Weighted frequencies were rounded up or down to integer values, as well as adjusted subpopulation sizes, which deviated across variables due to missing values. Missing values per variable and survey year can be found in the supplementary file.

### Inferential data analysis

First, logistic regression analyses for each survey year were done to assess the associations between the selected indicators and the outcome variable for each year separately. Finally, pooled logistic regression analyses, including data of all survey years, were carried out. Both the separate and pooled regression analyses were adjusted for urbanisation and region of residence to account for sample design elements instead of using the ‘svyset’ command that was used in the descriptive analyses. Design correction could not be done while analysing all survey data together, as each survey can only be accounted for by its own design.

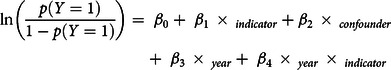




To assess the change over time of the studied associations, a multiple regression with interaction terms was used. The general equation of the models can be seen above, where the *β*_*1*_ represents the ‘base’ effect of the indicator. Specifically, this represents the effect of the studied indicator for the first survey year (2000). The *β*_*4*_ coefficient represents the interaction term between the survey year and the studied indicator. The interaction term quantifies the added change to the base effect in effect from a given year compared to the first year (2000). When the regression coefficient from one of the interaction terms is added to the base effect, the effect size of the particular year of interest can be calculated (as long as these are kept on their logarithmic scale). *P*-values of 0·05 or less were considered as statistically significant for all of the associations, and 95 % confidence intervals were reported for all estimates. Post-hoc margin plots were used to provide graphical depictions of the changes of associations.

### Ethical considerations

Data was derived from the Lao PDR MICS and LSIS surveys from UNICEF. Consent was asked for each interview and recorded in the datasets. For those who refused consent, no values were recorded on any of the questionnaire questions and they were excluded from any analyses. Datasets were accessed through correspondence with and consent of UNICEF, with the condition that datasets were used only for the intended and reported purposes and were not distributed. Finally, any reports made from the data were forwarded to UNICEF and discussed with the Lao government and stakeholders before dissemination or publication.

## Results

**Fig. 1:**
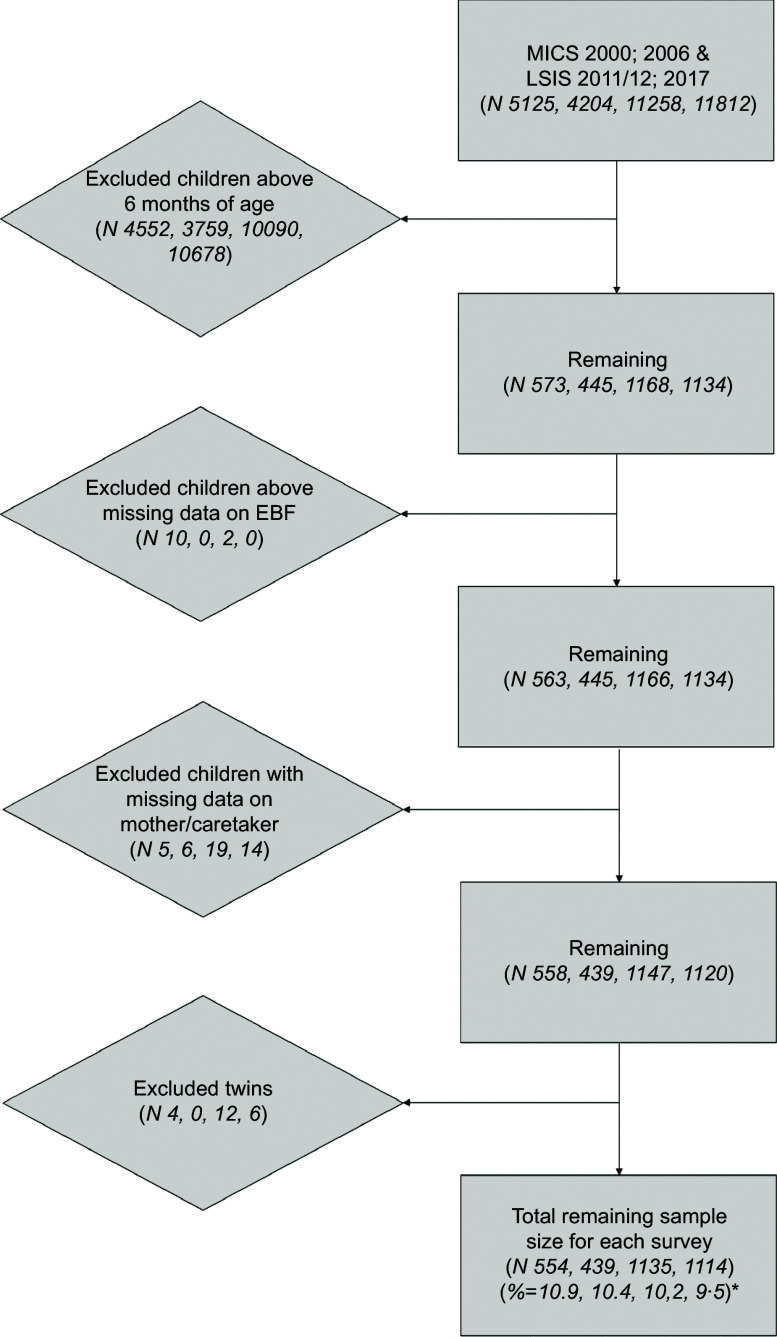
Flow chart for the application of exclusion criteria on the sample sizes (unweighted). *Percent of original sample size

After the application of the inclusion and exclusion criteria the analytical cohorts consisted of *N* 554, 439, 1135, 1114 (weighted frequencies: 558, 443, 1145, 1106) in the years 2000, 2006, 2011 and 2017, respectively, as seen in Table 1 and Fig. 1. These sample sizes comprised 10·8, 10·4, 10·1 and 9·4 % of the original sample sizes for the four years, respectively.

### Population characteristics

**Fig. 2:**
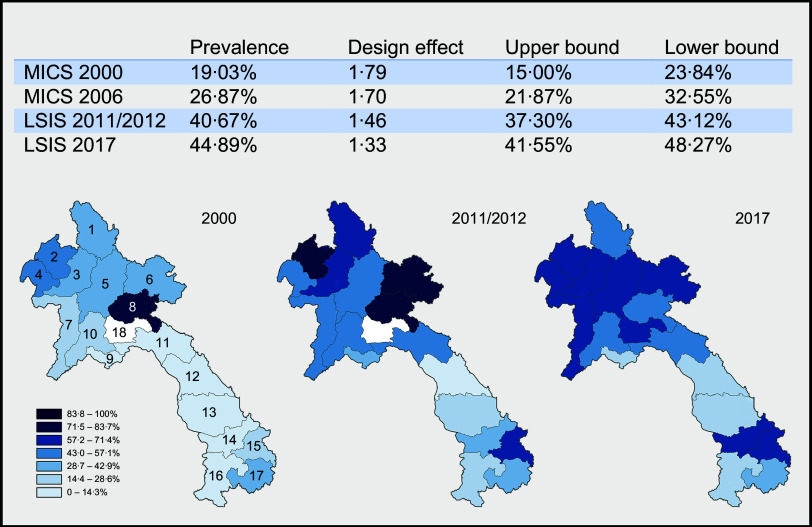
Prevalences per survey and geographical dispersion. Province 1: Phongsaly (30·77, 59·93 and 48·92 %), province 2: Luangnamtha (43·75, 73·80 and 64·09 %), province 3: Oudomxay (30·95, 61·69 and 65·51 %), province 4: Bokeo (43·48, 43·75 and 61·61 %), province 5: Luangprabang (34·09, 43·75 and 63·18), province 6: Huaphanh (32·35, 76·89 and 63·36 %), province 7: Sayabury (25·00, 55·82 and 65·52 %) and province 8: Xiengkhuang (76·00, 77·9 and 57·58 %) make up the Northern region of Lao PDR. Province 9: Vientiane Capital (12·50, 32·46 and 18·8 %), province 10: Vientiane Province (18·75, 55·83 and 55·49 %), province 11: Borikhamxay (10·00, 45·00 and 46·55 %), province 12: Khammuane (7·14, 14·55 and 17·00 %), province 13: Savannakhet (1·89, 16·16 and 18·14 %) make up the central region of Lao PDR. Finally, province 14: Saravane (1·72, 28·88 and 61·58 %), province 15: Sekong (13·04, 61·84 and 57·74 %), province 16: Champasack (0, 19·22 and 26·03 %) and province 17: Attapeu (29·17, 40·03 and 40·62) make up the Southern region of Lao PDR. Province 18: Xaysomboon (62·82 %) coloured in white in 2000 and 2011/2012 was a special municipality until 2013 and did not have any data on EBF prior to this

Adjusted rates of EBF were estimated at 19·03, 26·87, 40·67 and 44·89 % in the years 2000, 2006, 2011/2012 and 2017, respectively (see Fig. 2). The largest increase in EBF can be seen between the years 2006 and 2011/2012; this difference was significant as the respective confidence intervals did not overlap. Furthermore, the increase stagnated in the last year, rising by 4·22 % points, which was NS. Additionally, the design effect of the population estimates was between 1·33 and 1·79 with the lower values belonging to the recent survey years.

It can be observed that the majority of the sample populations were located in the central part of Lao PDR, especially in the most recent years (46·41 and 44·87 % in 2011/2012 and 2017, respectively). Across the samples, there was an increase observed in receivers of ANC, growing from 30·64 % in 2000 to 84·14 % in 2017. Additionally, the number of caretakers/mothers that received more than the WHO recommended number of ANC visits (four) increased from 27·23 % to 48·77 % between 2011/2012 and 2017. The proportion of children with mothers having secondary education rose from 14·17 % to 42·38 %, whereas the proportion of women having no education decreased from 42·64 % to 18·41 %. The proportion of mothers engaging in early initiation (time put to breast: immediately) of breast-feeding increased from 31·71 % to 48·10 %. Finally, the proportion of children with a vaccination card also increased from 30·77 % to 77·40 % over the years.

### Inferential statistics results

In the multivariate analyses per survey year (Table 2), various indicators were found to be significantly associated with EBF. Region was significantly associated with EBF across all survey years, showing significantly lower odds for both the Central (2000 OR: 0·51, CI 95 %: 0·30, 0·85; 2006 OR: 0·39, CI 95 %: 0·23 – 0·67; 2011 OR: 0·43, CI 95 %: 0·32, 0·57; 2017 OR: 0·42, CI 95 %: 0·31, 0·56) and Southern regions (2000 OR: 0·11, CI 95 %, 2006 OR: 0·09, CI 95 %: 0·05, 0·18; 2011 OR: 0·44, CI 95 %: 0·32, 0·59; 2017 OR: 0·55, CI 95 %: 0·84, 1·46) of Lao PDR compared with the North. This association also became evident from the geographical dispersion of EBF prevalence in the heat maps presented in Fig. 2. In the year 2017, children of caretakers/mothers that did not watch television were more likely to receive EBF, as caretakers/mothers that watched almost every day (OR: 0·69, CI 95 %: 0·52, 0·91), at least once a week (OR: 0·54, CI 95 %: 0·34, 0·83) and less than once a week (OR: 0·50, CI 95 %: 0·26, 0·98) all showed inverse relationship. Higher wealth status showed to be significantly negatively associated for the years 2011/2012 and 2017, specifically for the fourth (OR: 0·65, CI 95 %: 0·44, 0·98) and richest quintiles (OR: 0·57, CI 95 %: 0·35, 0·95) in 2011/2012 and also the fourth (OR: 0·58, CI 95 %: 0·38, 0·87) and richest quintile (OR: 0·39, CI 95 %: 0·25, 0·62) in 2017, compared with the poorest quintile. Ethnicity was a strong significant indicator across all survey years. All of the ethnic groups: the Lao-Thai, (2000 OR: 0·15, CI 95 %: 0·07, 0·33; 2006 OR: 0·31, CI 95 %: 0·15, 0·64; 2011 OR: 0·31, CI 95 %: 0·20, 0·46; 2017 OR: 0·38, CI 95 %: 0·26, 0·54), Mon-Khmer (2000 OR: 0·36, CI 95 %: 0·17, 0·75; 2011 OR: 0·52, CI 95 %: 0·32, 0·84; 2017 OR: 0·60, CI 95 %: 0·41, 0·88) and others (2000 OR: 0·22, CI 95 %: 0·10, 0·46; 2006 OR: 0·15, CI 95 %: 0·06, 0·37; 2011 OR: 0·42, CI 95 %: 0·28, 0·63) showed a significantly lower odds on EBF compared with the Hmong-Mien, except for the Mon-Khmer in 2006 and others groups in 2017. Religion was significantly associated in the last two survey years, showing significantly higher odds for children of caretakers/mothers with an animist household head (2011 OR: 1·87, CI 95 %: 1·44, 2·44; 2017 OR: 1·71, CI 95 %: 1·33, 2·23) to exclusively breastfeed their child compared with those with a Buddhist household head. A significant association was also found for children from female-headed households in 2011/2012 and 2017 (2011 OR: 2·89, CI 95 %: 1·13, 7·36; 2017 OR: 0·47, CI 95 %: 0·29, 0·77), the direction of which was, however, different for the two years.

**Table 2:** Adjusted logistic regression analyses for associated factor of exclusive breast-feeding of children between 0 and 5 months old per survey year

	MICS 2000		MICS 2006		LSIS 2011/2012		2017	
	OR	CI 95 %	OR	CI 95 %	OR	CI 95 %	OR	CI 95 %
Region (Ref = north)								
Central	0·51*	0·30, 0·85	0·39***	0·23, 0·67	0·43***	0·32, 0·57	0·42***	0·32, 0·56
South	0·11***	0·06, 0·23	0·09***	0·05, 0·18	0·44***	0·32, 0·59	0·55***	0·40, 0·75
Residence (Ref = urban)								
Rural	1·72	0·85, 3·48	1·43	0·72, 2·85	1·00	0·73, 1·36	1·11	0·84, 1·46
Frequency of watching TV of caretaker (Ref = not at all)								
Almost every day	–	–	–	–	0·82	0·62, 1·08	0·69**	0·52, 0·91
At least once a week	–	–	–	–	0·92	0·61, 1·40	0·54**	0·35, 0·83
Less than once a week	–	–	–	–	0·75	0·42, 1·34	0·50*	0·26, 0·98
Wealth index (Ref = poorest)								
Second	1·88	1·01, 3·50	1·19	0·64, 2·18	0·91	0·65, 1·27	0·99	0·71, 1·39
Middle	1·23	0·62, 2·43	1·14	0·55, 2·37	0·80	0·57, 1·14	0·91	0·63, 1·30
Fourth	0·65	0·31, 1·37	0·76	0·30, 1·92	0·65*	0·44, 0·98	0·58**	0·38, 0·87
Richest	0·70	0·29, 1·70	1·87	0·70, 5·01	0·57*	0·35, 0·95	0·39***	0·25, 0·62
Caretaker received ANC (Ref = no)								
Yes	0·73	0·41, 1·30	1·04	0·62, 1·76	1·00	0·77, 1·28	1·00	0·72, 1·39
Times received ANC (Ref = 0)								
1	–	–	–	–	0·98	0·57, 1·67	1·15	0·62, 2·13
2	–	–	–	–	1·26	0·77, 2·06	0·80	0·46, 1·38
3	–	–	–	–	0·74	0·47, 1·16	1·03	0·65, 1·63
4	–	–	–	–	1·26	0·81, 1·96	0·96	0·62, 1·49
< 4	–	–	–	–	0·95	0·68, 1·31	1·03	0·72, 1·46
Caretaker practiced food taboo (Ref = no)								
Yes	1·93	0·83, 4·50	0·99	0·50, 1·92	–	–	–	–
Place of birth (Ref = at home)								
Health facility	–	–	–	–	1·07	0·82, 1·39	0·79	0·61, 1·03
Gender of household head (Ref = male)								
Female	0·51	0·15, 1·78	2·89*	1·13, 7·36	0·47**	0·29, 0·77	0·70	0·46, 1·06
Ethnicity of household head (Ref = Hmong-Mien)								
Lao-Thai	0·15***	0·07, 0·33	0·31**	0·15, 0·64	0·31***	0·20, 0·46	0·38***	0·26, 0·54
Mon-Khmer	0·36**	0·17, 0·75	0·65	0·32, 1·33	0·52 **	0·32, 0·84	0·60 **	0·41, 0·88
Other groups	0·22***	0·10, 0·46	0·148***	0·06, 0·37	0·42***	0·28, 0·63	0·66	0·36, 1·20
Religion of household head (Ref = Buddhist)								
Animist	–	–	–	–	1·87***	1·44, 2·44	1·71***	1·33, 2·23
Other	–	–	3·75	0·92, 15·3	1·57	0·53, 4·60	2·45*	1·01, 5·96
No religion	–	–	1·68	0·92, 3·08	–	–	–	–
Gender of child (Ref = male)								
Female	0·85	0·54, 1·33	1·05	0·66, 1·67	1·27*	1·00, 1·62	1·18	0·93, 1·50
Child age in months (Ref = 0)								
1 month	0·55	0·25, 1·21	1·68	0·693, 4·087	0·83	0·56, 1·23	0·40***	0·25, 0·64
2 months	0·37*	0·17, 0·80	0·95	0·369, 2·422	0·58**	0·39, 0·86	0·39***	0·25, 0·60
3 months	0·31***	0·14, 0·71	0·48	0·188, 1·222	0·62*	0·41, 0·95	0·24***	0·15, 0·37
4 months	0·31***	0·14, 0·72	0·39	0·149, 1·023	0·27***	0·17, 0·41	0·15***	0·09, 0·23
5 months	0·13***	0·05, 0·35	0·17**	0·053, 0·524	0·21***	0·13, 0·32	0·06***	0·04, 0·10
Age of caretaker (Ref = < 20)								
20–24	1·10	0·55, 2·22	1·17	0·58, 2·34	0·99	0·70, 1·41	0·81	0·55, 1·17
25–29	1·10	0·51, 2·34	1·16	0·57, 2·34	1·06	0·73, 1·55	0·81	0·55, 1·18
30–34	0·64	0·27, 1·52	0·44	0·17, 1·12	1·09	0·71, 1·68	0·81	0·52, 1·27
35–39	1·61	0·71, 3·64	1·72	0·69, 4·26	0·66	0·39, 1·12	0·98	0·57, 1·66
40–44	1·44	0·46, 4·47	0·78	0·23, 2·57	0·96	0·49, 1·87	0·38*	0·16, 0·91
Marital status of caretaker (Ref = In union)								
Not in union	0·46	0·13, 1·62	–	–	0·36*	0·15, 0·88	0·56	0·22, 1·43
Did child have diarrhoea in preceding 2 weeks? (Ref = no)								
Yes	0·54	0·20, 1·48	0·35*	0·13, 0·96	0·47***	0·30, 0·74	0·36***	0·21, 0·62
Amount of children born by caretaker (Ref = 1)								
2	–	–	–	–	1·02	0·74, 1·40	1·07	0·78, 1·47
3	–	–	–	–	1·06	0·73, 1·54	1·25	0·88, 1·78
4	–	–	–	–	0·70	0·44, 1·13	1·62*	1·07, 2·47
< 4					0·97	0·69, 1·36	1·32	0·87, 2·01
Education of caretaker (Ref = none)								
Primary	0·79	0·48, 1·30	0·97	0·57, 1·66	0·70*	0·53, 0·94	1·09	0·79, 1·52
Secondary or higher	0·95	0·43, 2·12	1·49	0·70, 3·18	1·00	0·72, 1·38	0·91	0·65, 1·29
Reading ability of caretaker (Ref = Cannot read)								
Has difficulty	1·10	0·49, 2·46	1·13	0·49, 2·60	0·75	0·50, 1·12	1·28	0·84, 1·95
No difficulty	0·66	0·40, 1·08	0·93	0·48, 1·76	1·00	0·71, 1·40	0·96	0·65, 1·41
Time put baby to breast (Ref = Immediately)								
Hours	0·72	0·42, 1·22	0·73	0·39, 1·39	1·16	0·87, 1·53	0·87	0·66, 1·14
Days	0·65	0·34, 1·24	0·32***	0·18, 0·59	0·76	0·56, 1·04	0·66*	0·46, 0·93
Child has vaccination card (Ref = no)								
Yes	0·54*	0·31, 0·93	0·76	0·47, 1·25	0·73*	0·57, 0·94	0·75*	0·57, 1·00

Sample size per variable can be found in supplementary file.

*P < 0·05, **P < 0·01, ***P < 0·001.

A higher age of the child was negatively associated with exclusive breast-feeding; the odds for the child being exclusively breastfed decreased for each subsequent month of age (see Table 2). This association was significant for most of the survey years, with the exception of 2006, when the only significance was for children aged five months. It was notable that the odds-ratios for age in 2017 were the smallest, compared with the preceding years. For each year, except 2000, children who had diarrhoea in the two weeks preceding the interview had significantly lower odds of being exclusively breastfed compared with those who did not have diarrhoea (2006 OR: 0·35, CI 95 %: 0·13, 0·96; 2011 OR: 0·47, CI 95 %: 0·30, 0·74; 2017 OR: 0·36, CI 95 %: 0·21, 0·62). For the years 2006 and 2017, mothers who had put their most recently born child to the breast within days after birth had lower odds of exclusively breast-feeding their child, compared with those that did it immediately (2006 OR: 0·32, CI 95 %: 0·18, 0·59; 2017 OR: 0·66, CI 95 %: 0·46, 0·93). Additionally, in 2000 and 2011/2012, children who had a vaccination card were less likely to be exclusively breastfed than those who did not (2000 OR: 0·54, CI 95 %: 0·31, 0·93; 2011 OR: 0·73, CI 95 %: 0·57, 0·94; 2017 OR: 0·75, CI 95 %: 0·57, 1·00).

### Changes over time

The pooled analyses (Table 3) showed a significant change over time for ethnicity. As seen in the table, there is a positive increasing trend for all ethnic groups over the years, all odds ratios being above 1 except for the ‘others’ group in 2006. The Lao ethnic group saw a significant increase in the most recent years when compared with the year 2000 (2011 OR: 3·33, CI 95 %: 1·48, 7·50; 2017 OR: 4·15, CI 95 %: 1·87, 9·19). The group containing the ‘other’ ethnicities saw a similar increase, with two significant changes in the last two survey years (2011 OR: 2·98, CI 95 %: 1·33, 6·68; 2017 OR: 4·88, CI 95 %: 1·95, 12·23). Finally, the Mon-Khmer saw smaller positive changes, none of which were significant.

**Table 3:** Pooled logistic regression analyses with interaction terms for the development of associated factor of exclusive breast-feeding of children between 0 and 5 months old

	MICS 2000		MICS 2006		LSIS 2011/2012		LSIS 2017	
	OR	CI 95 %	OR	CI 95 %	OR	CI 95 %	OR	CI 95 %
Region (Ref = north)								
Central	0·47	0·28, 0·78	0·81	0·39, 1·70	0·84	0·47, 1·48	0·90	0·51, 1·61
South	0·110	0·06, 0·22	0·84	0·32, 2·19	3·96***	1·89, 8·29	4·99***	2·37, 10·49
Residence (Ref = urban)								
Rural	1·71	0·873, 3·36	0·79	0·31, 2·02	0·58	0·28, 1·23	0·66	0·32, 1·37
Frequency of watching TV of caretaker (Ref = not at all)								
Almost every day	–	–	–	–	0·83	0·63, 1·09	0·82	0·56, 1·21
At least once a week	–	–	–	–	0·93	0·62, 1·41	0·57	0·31, 1·04
Less than once a week	–	–	–	–	0·74	0·41, 1·33	0·68	0·28, 1·64
Wealth index (Ref = poorest)								
Second	1·71	0·95, 2·87	0·62	0·27, 1·39	0·53	0·27, 1·04	0·59	0·30, 1·16
Middle	0·97	0·51, 1·73	0·85	0·34, 2·14	0·84	0·40, 1·72	0·95	0·46, 1·97
Fourth	0·55	0·27, 1·03	1·09	0·36, 3·31	1·24	0·55, 2·78	1·07	0·48, 2·40
Richest	0·9	0·21, 1·04	2·05	0·69, 6·07	1·25	0·50, 3·16	0·81	0·32, 2·04
Caretaker received ANC (Ref = no)								
Yes	0·74	0·43, 1·25	1·30	0·65, 2·62	1·38	0·77, 2·45	1·32	0·71, 2·45
Times received ANC (Ref = 0)								
1	–	–	–	–	0·98	0·58, 1·67	1·53	0·78, 3·02
2	–	–	–	–	1·26	0·78, 2·05	1·05	0·56, 1·95
3	–	–	–	–	0·76	0·49, 1·19	1·62	0·94, 2·79
4	–	–	–	–	1·24	0·80, 1·90	0·83	0·49, 1·40
< 4	–	–	–	–	0·99	0·73, 1·34	1·17	0·80, 1·70
Caretaker practiced food taboo (Ref = no)								
Yes	1·99	0·86, 4·59	0·474	0·18, 1·27	–	–	–	–
Place of birth (Ref = at home)								
Health facility	–	–	0·85	0·49, 1·47	1·27	0·70, 2·31	0·91	0·5, 1·67
Gender of household head (Ref = male)								
Female	0·48	0·14, 1·61	3·91	0·90, 17·08	1·03	0·28, 3·83	1·53	0·42, 5·56
Ethnicity of household head (Ref = Hmong-Mien)								
Lao-Thai	0·1***	0·05, 0·20	2·37	0·93, 6·01	3·33**	1·48, 7·50	4·15***	1·87, 9·19
Mon-Khmer	0·34**	0·17, 0·70	1·84	0·67, 5·03	1·50	0·63, 3·56	2·02	0·90, 4·53
Other groups	0·15***	0·07, 0·30	0·68	0·23, 2·03	2·98**	1·33, 6·68	4·88***	1·95, 12·23
Religion of household head (Ref = Buddhist) †								
Animist	–	–	–	–	1·95	1·52, 2·50	0·81	0·60, 1·09
Other religion	–	–	–	–	1·60	0·55, 4·67	0·79	0·24, 2·62
Gender of child (Ref = male)								
Female	0·90	0·58, 1·39	1·23	0·66, 2·28	1·43	0·87, 2·35	1·31	0·80, 2·15
Age of child (Ref = 0)								
1 month	0·53	0·25, 1·14	3·06	1·00, 9·41	1·56	0·66, 3·67	0·74	0·90, 1·80
2 months	0·35	0·17, 0·73	2·82	0·90, 8·85	1·61	0·70, 3·74	1·09	0·46, 2·57
3 months	0·30	0·14, 0·66	1·80	0·56, 5·85	2·08	0·86, 5·04	0·80	0·33, 1·97
4 months	0·31	0·14, 0·68	1·37	0·41, 4·53	0·85	0·34, 2·11	0·49	0·20, 1·22
5 months	0·13	0·05, 0·34	1·43	0·33, 6·19	1·54	0·53, 4·48	0·46	0·15, 1·33
Age of caretaker (Ref = < 20)								
20–24	1·02	0·52, 2·02	0·98	0·38, 2·53	0·97	0·45, 2·10	0·79	0·36, 1·72
25–29	0·91	0·44, 1·90	1·15	0·43, 3·10	1·19	0·52, 2·71	0·89	0·39, 2·05
30–34	0·61	0·26, 1·43	0·69	0·20, 2·40	1·83	0·70, 4·76	1·37	0·53, 3·57
35–39	1·45	0·66, 3·18	1·10	0·34, 3·51	0·47	0·18, 1·20	0·70	0·27, 1·81
40–44	1·25	0·42, 3·72	0·58	0·12, 2·82	0·80	0·22, 2·87	0·31**	0·08, 1·25
Marital status of caretaker (Ref = in union)								
Not in union	0·46	0·13, 1·56	–	–	0·78	0·17, 3·55	1·28	0·27, 6·00
Child had diarrhoea in the preceding 2 weeks? (Ref = no)								
Yes	0·58	0·22, 1·57	0·60	0·15, 2·38	0·77	0·26, 2·28	0·59	0·19, 1·83
Children ever born by caretaker (Ref = 1)								
2	–	–	–	–	1·02	0·74, 1·40	0·81	0·55, 1·20
3	–	–	–	–	1·04	0·72, 1·51	0·94	0·60, 1·47
4	–	–	–	–	0·69	0·43, 1·11	1·74*	1·00, 3·02
< 4	–	–	–	–	0·93	0·67, 1·30	1·07	0·69, 1·66
Education (Ref = none)								
Primary	0·69	0·43, 1·11	1·15	0·58, 2·28	1·04	0·60, 1·81	1·61	0·90, 2·87
Secondary or higher	0·78	0·39, 1·58	1·34	0·53, 3·39	1·34	0·61, 2·79	1·14	0·53, 2·47
Reading ability of caretaker (Ref = Cannot read)								
Has difficulty	0·85	0·40, 1·81	1·15	0·39, 3·37	0·93	0·39, 2·19	1·59	0·66, 3·84
No difficulty	0·55*	0·34, 0·89	1·23	0·58, 2·61	1·80*	1·00, 3·23	1·86*	1·01, 3·41
Time put baby to breast (Ref = immediately)								
Hours	0·71	0·43, 1·17	0·98	0·46, 2·10	1·60	0·90, 2·85	1·17	0·66, 2·07
Days	0·52*	0·29, 0·93	0·64	0·28, 1·43	1·48	0·76, 2·87	1·24	0·64, 2·50
Child has vaccination card (Ref = no)								
Yes	0·54*	0·32, 0·91	1·31	0·66, 2·62	1·37	0·77, 2·44	1·37	0·75, 2·48

Sample size per variable can be found in supplementary file.

*P < 0·05, **P < 0·01, ***P < 0·001.

† Religion in 2006 was excluded, as animism was possibly documented as ‘no religion’ although this was not certain.

**Fig. 3:**
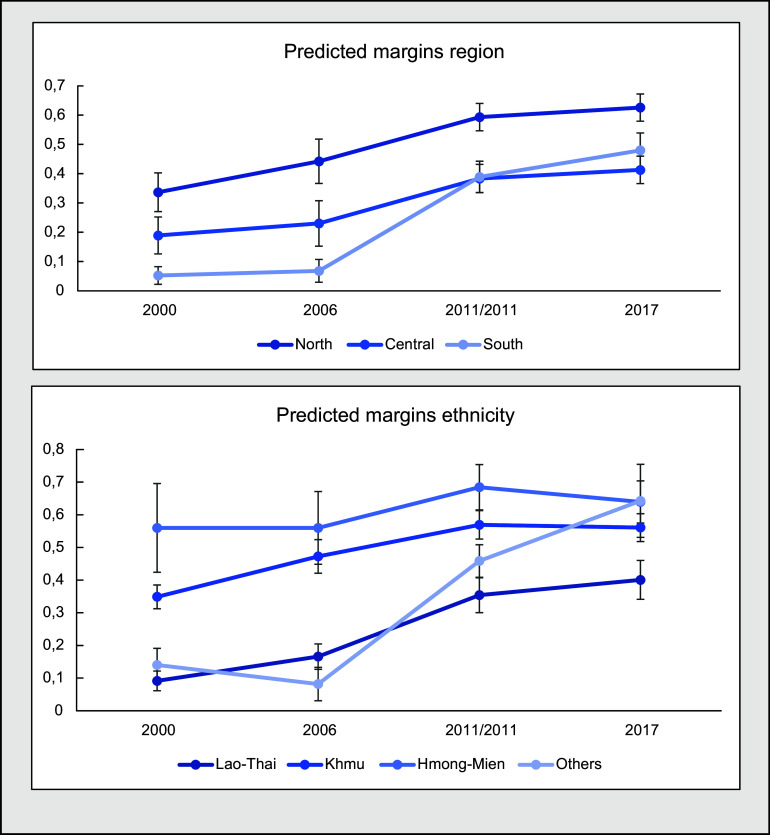
Predicted margins of pooled multivariate regression analyses

The other significant change that was found through the analysis was for region, the mechanisms of which are visible in the heat maps. From the results it can be seen that the Southern part of Lao PDR had improved significantly in the last two survey years (2011 OR: 3·96, CI 95 %: 1·89, 8·29; 2017 OR: 4·99, CI 95 %: 2·37, 10·49), whereas the central region had not improved at all, compared with the North. The changes in both associations can be seen by the predicted margin proportions in Fig. 3.

## Discussion

Using the rich data from the MICS and LSIS, we showed that the rates of EBF rose from 19·03 to 26·87 to 40·67 and finally to 44·89 % in the respective years: 2000, 2006, 2011/2012 and 2017. The increase in EBF rate between the succeeding years was statistically significant, but with the development between 2011 and 2017 showing a stagnation of this increase. This development in EBF rates follows a similar trend reported by other countries, both in and outside of South-East Asia, hinting at global trends of EBF^(27)^.

Our analysis of temporality of associated factors revealed that the region of residence was strongly associated with EBF and also changed over time. Over the years, it can be observed that the mountainous northern region of Lao PDR had the highest EBF rates, but the Southern region of the country made efforts to close the gap between there and the North, surpassing the Central region. Furthermore, when we compare our prevalence maps in Fig. 2 with a geographical map of Laos, it becomes quite evident that mountainous areas – which make up the majority of Northern Lao PDR – had higher EBF rates.

Additionally, ethnicity of respondents played an important role in the rate of EBF. Of the changes found in association of ethnic group with EBF, the Lao-Thai in particular was observed to be closing the gap and showing an increased compliance with EBF. Ethnicity in Lao PDR explains various disparities between individuals. For example, individuals from the Lao-Thai ethnic group are generally well educated and wealthier when compared with the poorer and lesser educated Hmong-Mien, who often live in the more mountainous areas of Laos^(35,36)^. Additionally, different gender roles and post-partum practices can be found between ethnic groups, possibly explaining differences in adherence to breast-feeding practices between groups^(37)^. Implications can be drawn from the fact that the arena of policy making and scientific research is primarily dominated by the educated Lao-Thai. Policy changes may be most favourable to the Lao-Thai, since they are formulated primarily by people from this group. This is similar to the results found by Santos *et al*., in which EBF compliance increased most among wealthier mothers^(25)^. These findings align with the inverse equity hypothesis, which states that new health policy is generally picked up by wealthier populations first^(38)^. In the case of Lao PDR, this is most likely the result of disparities between ethnic groups in terms of wealth, education and cultural practices, and therefore of opportunities to implement EBF, and serves as a reminder that one size may not fit all when formulating policy^(39)^.

Although there is much overlap between ethnicity and region of residence, the analysis for ethnicity – which was corrected for region of residence – still explained disparities between EBF rates. This shows that region and ethnicity do not simply explain the same differences in EBF, even though the two factors are related.

In the setting of rapid development, change can also fail to take place, even where it would be expected. An example from the current study is the association with ANC visits, with which no association was established nor did this develop in the later years, despite the rapid upscaling of ANC to near-universal coverage (approaching 90 %). This finding coincided with the inverse association between the ownership of a vaccination card and EBF. Vaccination cards are often acquired from ANC visits and therefore may be a proxy for access of care, but not necessarily quality of care. Available research on the quality of (antenatal) care in Lao-PDR shows that it is often very poor, as health providers, among other issues, often lack adequate communication skills and provide insufficient health education^(40,41)^. This also becomes evident from the meagre 1·9 % of hospitals in Lao PDR that were Baby-Friendly Hospital Initiative certified in 2014^(42)^. The impact of the apparent lack of adequate ANC is also evident from other studies carried out in Lao PDR, showing that mothers often lack the knowledge on the specifics and benefits of EBF^(43,44)^. This has important implications as there has been some evidence that although deeply rooted traditional practices such as food taboos are in place, women from the Khmu ethnic group who were in strong engagement with health care showed some behaviour changes^(45)^.

### Strengths and weaknesses

The current study had several strengths and limitations worth noting. To our knowledge, no study of this kind has been carried out within the context of Lao-PDR, in which (recent) research on EBF is already scarce. Additionally, while there is a considerable amount of research on the trends and associated factors of EBF, very few studies have quantified and tested statistically how associations change over time. The present study succeeded in all three of these components, with the use of highly representative survey data from four years over a period of two decades in Lao PDR.

Given the standardised nature of health survey questionnaires, such as the MICS/LSIS, aggregation of data was relatively simple and valid. Another strength is that, due to the use of the MICS and LSIS survey data, fairly large sample sizes were achieved to arrive at high statistical power for the inferences made. By far and large variables were measured similarly across survey years, which supported aggregation and comparison. However, some variables were added or omitted over time. For example, practicing food taboos was part of the survey in 2000 and 2006, but omitted in the following years, while on the contrary, number of ANC visits was only added from 2011 onward. Also, categories of some variables changed over time. For example, in 2006, a variable consisted of categories ‘rural’ or ‘urban’, which was expanded in subsequent years to ‘urban’, ‘rural with road’ and ‘rural without road’. While variables can be collapsed into fewer categories, and thus allow for comparison, it inevitably leads to loss of information.

As with most studies using standardised survey data, there are limitations with respect to the use of 24 h recall questions, which is also the case for EBF. The nature of this measurement means that we know the rate of mothers practicing EBF at the time of the survey, but not the rate of mothers completing the full recommended six months of EBF. Thus, the estimated rate of EBF in these studies will be an overestimation of the women who complete the six months. This is also illustrated by the decline of EBF rates with increased age of infants.

Furthermore, complex sampling design corrections can only be accounted for one-at-a-time, therefore limiting the inferential analysis to using the shared sampling variables instead of the design correction, inevitably leading to a loss of statistical power. In this process, the estimation of numerous parameters also leads to another limitation of the current study: multiple testing. Considering the numbers of estimates and joint hypothesis tests conducted and due to the very nature of hypothesis testing, it is important to realise that multiple tests might lead to alpha-inflation. While it is not common practice to adjust for multiple comparisons in regression settings, it may be important to acknowledge and consider^(46)^.

Because of the strong data set, detailed analysis and minor limitations, the current study provides considerable evidence for changes in EBF practice. Based on representative data of Lao PDR, and the efforts made in the sampling designs, careful inferences can be made. Furthermore, the body of evidence surrounding EBF in Lao PDR has been mostly limited to cross-sectional studies, whereas the current study takes into account the missing temporal aspect of associations.

### Recommendations

We believe that the evidence gained from studies utilising (quasi) longitudinal study designs is important in the steering of policy making. This methodology allows us to track changes in associations over time, rather than isolated in separate years. The apparent benefit of this becomes clear through our study: we showed that increases in EBF rates were especially significant for the Lao-Thai, closing the gap with other ethnic groups, even though they practiced EBF the least overall. The latter would have likely been the conclusion of a cross-sectional study. This information enables policy makers to act on trends, rather than a snapshot.

With the wide availability of rich, routinely collected survey data, we recommend that such studies are carried out in addition to cross-sectional studies. Also, to ensure the validity and reproducibility of these studies, we would like to encourage investigation into which types of analyses are most applicable in these cases. For instance, the current study initially aimed to utilise a mixed-model analysis approach, but this idea was abandoned due to the small cluster sample sizes. Such analyses may be more parsimonious with respect to parameter estimation, and therefore provide analyses with higher statistical power. Whether such analyses provide more valid and robust results should be the subject to future research.

Besides the statistical considerations, the limitation with regard to the standardised measurement of EBF calls for alternative ways of measuring EBF. The recommendation is that mothers practice EBF for a six-month period, but the current methodology of measurement does not help us to assess this. Thus, inferences made from studies using the current methodology of measurement may not be entirely compatible with the goal we are trying to achieve. This is especially true since most evidence points towards the added benefit of practicing EBF for six months, particularly in low-income settings^(47,48)^. Finally, it also leaves us with the question of what the true rate of mothers is who complete the full six months of EBF. The need for such ways of measurement can potentially be satisfied with qualitative studies. Such studies can also provide explanations with respect to the established associations of the present and other studies, as well as providing new hypotheses for future studies.

The findings of the current study further emphasise the importance of and need for improved quality of care. The concerted efforts in the early 2000s have shown their successes, but have to be pushed forward so that the momentum is kept in improving EBF. Due to the discrepancies between ethnic groups and regions, special attention should be given to diversifying these initiatives so that they align with the goals and needs of the diverse population of Lao PDR, especially within the setting of ANC.

## Conclusion

The transitioning countries, among which Lao PDR is one, are in a stage of rapid development in all facets of society. These developments, both spatial and temporal, inevitably lead to changes in human behaviour, and in turn, to changes in phenomena such as EBF. When considering the formulation of policy and law, such developments need to be taken into account. The current study applied an easily replicable methodology that allows for the scrutiny of the kind. Finally, in the context of Lao PDR, the current study showed temporal discrepancies between regions and ethnicities, emphasising the need for appropriately designed interventions to improve EBF. These interventions are of special importance in the setting of ANC, as it is often the first and most important moment for EBF education for the mother and is currently lacking in Lao PDR.

## Supplementary material

For supplementary material accompanying this paper visit https://doi.org/10.1017/S1368980022001380. click here to view supplementary material
