# Supplementary material for: How do the determinants of exclusive breast-feeding change over time? A multi-survey quasi-longitudinal study in Lao People’s Democratic Republic
Source: Public Health Nutr. 2022 Jun 3;25(9):2380–94. doi: 10.1017/S1368980022001380 (PMC9991563; doi:10.1017/S1368980022001380)
Supplement: Supplementary file 1 [file S1368980022001380sup.zip › S1368980022001380sup001.docx]

**Supplementary file**

Table 1: Selected indicator variables with dataset of origin, survey year & aggregated variable labels

| Variable name | Dataset of origin | 2000 | 2006 | 2011/12 | 2017 | Aggregated value labels |
| --- | --- | --- | --- | --- | --- | --- |
| Total sample size | **CU5** | **5125** | **4204** | **11258** | **11812** | **-** |
| Region | CU5 | ✓ | ✓ | ✓ | ✓ | 1: North  2: Middle  3: South |
| Urbanization | CU5 | ✓ | ✓ | ✓ | ✓ | 1: Urban  2: Rural |
| Frequency of watching TV of caretaker | Women |  |  | ✓ | ✓ | 1: Almost everyday  2: Once a week  3: Less than once a week  4: Not at all |
| Wealth Index | CU5 | ✓ | ✓ | ✓ | ✓ | 1: Poorest  2: Second  3: Middle  4: Fourth  5: Richest |
| Caretaker received ANC | Women | ✓ | ✓ | ✓ | ✓ | 1: Yes  2: No |
| Times received ANC | Women |  |  | ✓ | ✓ | 5: > 4 times |
| Place of birth* | Women |  | ✓ | ✓ | ✓ | 1: Health facility  2: at home |
| Caretaker practiced food taboo | Women | ✓ | ✓ |  |  | 1: Yes  2: No |
| Sex of household head | Household | ✓ | ✓ | ✓ | ✓ | 1: Male  2: Female |
| Household size | Household | ✓ | ✓ | ✓ | ✓ | 11: > 10 |
| Ethnicity of household head | CU5/Household | ✓ | ✓ | ✓ | ✓ | 1: Lao-Thai  2: Mon-Khmer  3: Hmong Mien  4: Other/miss/DK |
| Religion of household head | CU5/Household |  | ✓ | ✓ | ✓ | 1: Buddhist  2: Animist  3: Other  4: No religion |
| Sex of child | CU5 | ✓ | ✓ | ✓ | ✓ | 1: Male  2: Female |
| Age of child in months | CU5 | ✓ | ✓ | ✓ | ✓ | 0-5 |
| Age of caretaker | Women | ✓ | ✓ | ✓ | ✓ | 1: 19-24 2: 25-29  3: 30-34 4: 35-39  5: 40-44 6: 45-49 |
| Caretaker currently in union | Women | ✓ |  | ✓ | ✓ | 1: Yes  2: No |
| Child had diarrhea in the past 2 weeks | CU5 | ✓ | ✓ | ✓ | ✓ | 1: Yes  2: No |
| Children ever born by caretaker | Women |  |  | ✓ | ✓ | 5: > 4 |
| Education of caretaker | CU5 | ✓ | ✓ | ✓ | ✓ | 1: None  2: Primary  3: Secondary or higher |
| Reading ability of caretaker | Women | ✓ | ✓ | ✓ | ✓ | 1: Cannot read  2: With difficulty  3: No problem |
| Time put baby to the breast* | Women | ✓ | ✓ | ✓ | ✓ | 1: Immediately  2: Hours  3: Days |
| Child has vaccination card | CU5 | ✓ | ✓ | ✓ | ✓ | 1: Yes  2: No |

**: These variables regarded the birth and breastfeeding of the most recently born child of the mother in question. Therefore, this means that it may not regard the child to which the variable is tied; as some mothers may have two children under 6 months of age.*

Table 2: Respective dataset sample sizes (unweighted)

|  | MICS 2000 | MICS 2006 | LSIS 2011/2012 | LSIS 2017 |
| --- | --- | --- | --- | --- |
| Children-under-5 | 5125 | 4204 | 11258 | 11812 |
| Women | 3633 | 7703 | 23937 | 26088 |
| Households | 6449 | 5995 | 19960 | 23299 |

Note: frequencies are unweighted

*Table 3: Proportions (%) of EBF per month of age of the child (weighted)*

|  | MICS 2000 | MICS 2006 | LSIS 2011/2012 | LSIS 2017 |
| --- | --- | --- | --- | --- |
| 0 months | 38.74 | 33.54 | 54.85 | 71.25 |
| 1 months | 23.49 | 43.33 | 47.92 | 49.68 |
| 2 months | 17.70 | 36.64 | 38.02 | 49.63 |
| 3 months | 17.02 | 22.40 | 40.75 | 42.05 |
| 4 months | 17.57 | 17.06 | 28.11 | 36.14 |
| 5 months | 8.80 | 9.69 | 24.78 | 17.23 |

**Data labelling tables**

### 2000

In the 2000 dataset, the women ID is composed of multiple variables in the following order: province (2 digit no), district number (2 digit no), cluster no (3 digit no), household number (3 digit no) and finally a line/unit number (2 digit no). See example below.

A women may have the following identifiers:

- Province: 1
- District: 2
- Cluster: 120
- Household: 80
- Line: 4

The women unique identifier will be: 01 20 120 080 04

### 2006, 2011/2012 and 2017

In the 2006 dataset, there are no recorded district and regions, therefore the unique identifier can only be made by using the cluster, household and line numbers provided. The same structure was found in the LSIS data of both years 2011/2012 and 2017.

**Outcome aggregation variables**

*Table 4: Outcome variable relevant variables*

| **MICS 2000** | **MICS 2006** | **LSIS 2011/2012** | **LSIS 2017** |
| --- | --- | --- | --- |
| Received: plain water (bf3b) | Child received plain water (BF3B) | Child drank plain water yesterday (BF3) | Child drank anything from a bottle with a nipple yesterday (BD4) |
| Received: sweetened water/juice (bf3c) | Child received sweetened water or juice (BF3C) | Child drank infant formula yesterday (BF4) | Child drank plain water yesterday (BD7A) |
| Received: milk (bf3e) | Child received infant formula (BF3E) | Child drank milk yesterday (BF6) | Child drank juice or juice drinks yesterday (BD7B) |
| Received: other liquids (bf3f) | Child received milk (BF3F) | Child drank juice or juice drinks yesterday (BF8) | Child drank clear broth / watery soup (nam keng) yesterday (BD7C) |
| Received: mushy food (bf3g) | Child received soya milk (BF3F1) | Child drank soup yesterday (BF9) | Child drank infant formula (cerelac, pediasure) yesterday (BD7D) |
| Received: rice (bf3h) | Child received soft or mushy food (BF3G) | Child drank any other liquid yesterday (BF12) | Child drank milk from animals (fresh, tinned, or powdered) yesterday (BD7E) |
| Received: meat (bf3i) | Child received semi solid or solid food (BF3H) | Child drank or ate yogurt yesterday (BF13) | Child drank any other liquid yesterday (BD7X) |
| Received: egg (bf3j) | Child received soft/mushy, semi solid or solid food (BF3GH) | Child ate thin porridge yesterday (BF15) | Child ate yogurt made from animal milk yesterday (BD8A) |
| Received: fish (bf3k) |  | Child ate solid or semi-solid food yesterday (BF16) | Child ate fortified baby food (cerelac, nestum, pediasure) yesterday (BD8B) |
| Received: fruit/vegetable (bf3l) |  | Child drank anything else from the bottle with a nipple yesterday (BF18) | Child ate bread, rice, noodles, porridge, or other foods made from grains yesterday (BD8C) |
| Received: fat and oil (bf3m) |  |  | Child ate pumpkin, carrots, squash or sweet potatoes that are yellow or orange inside yesterday (BD8D) |
|  |  |  | Child ate white potatoes, white yams, cassava or other foods made from roots yesterday (BD8E) |
|  |  |  | Child ate any dark green, leafy vegetables, such as spinach, morning glory, salad green, and green leaf lettuce yesterday (BD8F) |
|  |  |  | Child ate ripe mangoes, ripe papayas, or other locally available vitamin A-rich fruits yesterday (BD8G) |
|  |  |  | Child ate any other fruits or vegetables such as watermelon, banana yesterday (BD8H) |
|  |  |  | Child ate liver, kidney, heart or other organ meats yesterday (BD8I) |
|  |  |  | Child ate any other meat, such as beef, pork, lamb, goat, chicken, duck or sausages made from these meats yesterday (BD8J) |
|  |  |  | Child ate eggs yesterday (BD8K) |
|  |  |  | Child ate fresh or dried fish or shellfish yesterday (BD8L) |
|  |  |  | Child ate beans, peas, lentils or nuts or any food made from these yesterday (BD8M) |
|  |  |  | Child ate cheese or other food made from animal milk yesterday (BD8N) |
|  |  |  | Child ate other solid, semi-solid or soft food yesterday (BD8X) |

**Children’s dataset identifying variables**

*Table 5: Children’s dataset identifying variables*

| **Variables of interest** | **MICS 2000** | **MICS 2006** | **LSIS 2011-2012** | **LSIS 2017** | **Aggregated variable name** |
| --- | --- | --- | --- | --- | --- |
| **Province** | Hi7b |  | HH7 | HH7 | Province |
| **District** | Hi7a |  |  |  | District |
| **Cluster number** | Hi1 | UF1 | UF1 | UF1 | Cluster |
| **Household number** | Hi2 | UF2 | UF2 | UF2 | Household |
| **Line number of caretaker** | chctno | UF6 | UF6 | UF4 | Caretaker |
| **Sample weights** | Chweight | Chweight | Chweightq | Chweight | chweight |

**Missing values per variable MICS 2000**

Total cases in the set is 5125 (unweighted).

4552 cases excluded after applying age limit, leaving 573 cases.

10 cases had a missing value on the outcome variable, leaving 563 cases for the analysis.

Cases that had no data available on the caretaker/mother were also excluded, meaning that the caretaker was not present in the dataset at all, or there were missing values on every single variable for that specific caretaker/women. Excluding these cases, which amounted to 5 cases, leaving 558 cases.

Finally, any twins were excluded from the analyses as well, which amounted to 4 cases, leaving 554 for the analyses.

*Table 6: Missing values per variables MICS 2000*

| **Variable names** | **Missing cases** | **Valid N** |
| --- | --- | --- |
| Sex | 0 (0%) | 554 (100%) |
| Child had diarrhea | 1 (<1%) | 553 (>99%) |
| Residence (U/R) | 0 (0%) | 552 (100%) |
| Education of mother | 4 (<1%) | 550 (>99%) |
| Wealth Index | 0 (0%) | 554 (100%) |
| Ethnicity of household head | 0 (0%) | 554 (100%) |
| Religion | Not in set | Not in set |
| **Women variables** |  |  |
| Womens age | 0 (0%) | 554 (100%) |
| Food Taboo | 2 (<1%) | 552 (>99%) |
| Place of birth | Not in set | Not in set |
| Current marital status | 0 (0%) | 554 (100%) |
| Owns a TV | 3 (<1%) | 551 (>99%) |
| Frequency of watching TV | Not in set | Not in set |
| Reading ability | 7 (1.25%) | 547 (98.75%) |
| Children ever born | Not in set | Not in set |
| Children ever dead | Not in set | Not in set |
| Received antenatal care | 95 (17.15%) | 459 (82.85%) |
| Frequency reading newspaper | Not in set |  |
| Time put baby to the breast | 29 (5.23%) | 525 (94.77%) |
| **Household variables** |  |  |
| Household size | 0 (0%) | 554 (100%) |
| Sex of household head | 0 (0%) | 554 (100%) |
| Age of household head | 1 (<1%) | 553 (>99%) |

Note: all frequencies are unweighted

**The total weighted population was 548.99 or 549 cases**

**Missing values per variable MICS 2006**

Total cases in the set is 4204 (unweighted).

3759 cases were excluded after applying the age filter, leaving 445 cases.

There were no missing values on the outcome variable, leaving the 445 cases.

Cases that had no data available on the caretaker/mother were also excluded, meaning that the caretaker was not present in the dataset at all, or there were missing values on every single variable for that specific caretaker/women. Excluding these cases, which amounted to 6 cases left 439 for the analyses.

Finally, any twins were excluded from the analyses as well, which amounted to 0 cases, leaving 439 for the analyses.

*Table 7: Missing values per variables MICS 2006*

| **Variable names** | **Missing cases** | **Valid N** |
| --- | --- | --- |
| Sex | 0 (0%) | 439 (100%) |
| Child had diarrhoea | 0 (0%) | 439 (100%) |
| Residence (U/R) | 0 (0%) | 439 (100%) |
| Education of mother | 2 (<1%) | 437 (>99%) |
| Wealth Index | 0 (0%) | 439 (100%) |
| Ethnicity of household head | 0 (0%) | 439 (100%) |
| Religion | 0 (0%) | 439 (100%) |
| **Women variables** |  |  |
| Womens age | 0 (0%) | 439 (100%) |
| Food taboo | 9 (2.05%) | 430 (97.95%) |
| Place of birth | 9 (2.05%) | 430 (97.95%) |
| Current marital status | Not in this set | Not in this set |
| Owns a TV | 0 (0%) | 439 (100%) |
| Frequency of watching TV | 0 (0%) | 439 (100%) |
| Owns a radio | 0 (0%) | 439 (100%) |
| Reading ability | 88 (20.05%) | 351 (79.95%) |
| Children ever born | Not in this set | Not in this set |
| Children ever dead | Not in this set | Not in this set |
| Received antenatal care | 0 (0%) | 439 (100%) |
| Time put baby to breast | 12 (2.73%) | 427 (97.27%) |
| **Household variables** |  |  |
| Household size | 0 (0%) | 439 (100%) |
| Sex of household head | 0 (0%) | 439 (100%) |
| Age of household head | Not in set | Not in set |

Note: all frequencies are unweighted

**The total weighted population size amounted to 443.24 or 443 rounded down**

**Missing values per variable LSIS 2011/2012**

Total cases in this set is 11258 (unweighted).

10090 cases were excluded after applying the age filter, leaving 1168 cases

There were 2 cases with a missing values on the outcome variable, leaving 1166 cases for the final analysis.

Cases that had no data available on the caretaker/mother were also excluded, meaning that the caretaker was not present in the dataset at all, or there were missing values on every single variable for that specific caretaker/women. Excluding these cases, which amounted to 19 cases leaving 1147 for the final analysis.

Finally, any twins were excluded from the analyses as well, which amounted to 12 cases, leaving 1135 for the analyses.

*Table 8: Missing values per variables MICS 2011/2012*

| **Variable names** | **Missing cases** | **Valid N** |
| --- | --- | --- |
| Sex | 0 (0%) | 1135 (100%) |
| Child had diarrhea | 0 (0%) | 1135 (100%) |
| Residence (U/R) | 0 (0%) | 1135 (100%) |
| Education of mother | 0 (0%) | 1135 (100%) |
| Wealth Index | 0 (0%) | 1135 (100%) |
| Ethnicity of household head | 0 (0%) | 1135 (100%) |
| Religion | 2 (<1%) | 1133 (>99%) |
| **Women variables** |  |  |
| Women’s age | 0 (0%) | 1135 (100%) |
| Food taboo | Not in this set | Not in this set |
| Place of birth | 8 (<1%) | 1127 (>99%) |
| Caretaker/mothers age | 0 (0%) | 1135 (100%) |
| Current marital status | 0 (0%) | 1135 (100%) |
| Owns a TV | 0 (0%) | 1135 (100%) |
| Frequency of watching TV | 0 (0%) | 1135 (100%) |
| Owns a radio | 479 (42.20%) | 656 (57.80%) |
| Reading ability | 341 (30.04%) | 794 (69.96%) |
| Children ever born | 0 (0%) | 1135 (100%) |
| Children ever dead | 0 (0%) | 1135 (100%) |
| Received antenatal care | 8 (<1%) | 1127 (>99%) |
| Time put baby to breast | 31 (2.73%) | 1104 (97.27%) |
| **Household variables** |  |  |
| Household size | 0 (0%) | 1135 (100%) |
| Sex of household head | 0 (0%) | 1135 (100%) |

Note: all frequencies are unweighted

**The total weighted population size amounts up to 1145,24 or 1145 rounded down**

**Missing values per variable LSIS 2017**

Total cases in this set is 11812

10678 cases were excluded after applying the age filter leaving 1134 cases

There were no cases with a missing value on the outcome variable, leaving all 1134 cases to analyze.

Cases that had no data available on the caretaker/mother were also excluded, meaning that the caretaker was not present in the dataset at all, or there were missing values on every single variable for that specific caretaker/women. Excluding these cases, which amounted to 14 cases leaving 1120 for the analyses.

Finally, any twins were excluded from the analyses as well, which amounted to 6 cases, leaving 1114 for the analyses.

*Table 9: Missing values per variables MICS 2017*

| **Variable names** | **Missing cases** | **Valid N** |
| --- | --- | --- |
| Sex | 0 (0%) | 1114 (100%) |
| Child had diarrhea | 3 (<1%) | 1111 (>99%) |
| Residence (U/R) | 0 (0%) | 1114 (100%) |
| Education of mother | 0 (0%) | 1114 (100%) |
| Wealth Index | 0 (0%) | 1114 (100%) |
| Ethnicity of household head | 0 (0%) | 1114 (100%) |
| Religion | 0 (0%) | 1114 (100%) |
| **Women variables** |  |  |
| Women’s age | 0 (0%) | 1114 (100%) |
| Food taboo | Not in this set | Not in this set |
| Place of birth | 19 (1.71%) | 1095 (98.29%) |
| Current marital status | 0 (0%) | 1114 (100%) |
| Owns a TV | 1 (<1%) | 1113 (>99%) |
| Frequency of watching TV | 1 (<1%) | 1113 (>99%) |
| Owns a radio | 0 (0%) | 1114 (100%) |
| Reading ability | 462 (41.47%) | 652 (58.53%) |
| Children ever born | 0 (0%) | 1114 (100%) |
| Children ever dead | 0 (0%) | 1114 (100%) |
| Received antenatal care | 18 (1.62%) | 1096 (98.38%) |
| Time put baby to breast | 32 (2.69%) | 1084 (97.31%) |
| **Household variables** |  |  |
| Household size | 0 (0%) | 1114 (100%) |
| Sex of household head | 0 (0%) | 1114 (100%) |

Note: all frequencies are unweighted

**The total weighted population size amounts to 1107,72 or 1108 rounded up**
